# Supplementary material for: Are carbon emissions trading and green financial instruments synergistic? -Comprehensive quantitative research based on content analysis
Source: PLoS One. 2024 Mar 7;19(3):e0298601. doi: 10.1371/journal.pone.0298601 (PMC10919601; doi:10.1371/journal.pone.0298601)
Supplement: S1 Data — (ZIP) [file pone.0298601.s001.zip › Data/地级市Z_F工作报告.zip!/╣π╬≈/╣≤╕█╩╨/└┤╫╘╣1⁄2╓┌║┼í╛┬φ┐╦╩2╛▌═°í┐_2011.docx]

各位代表：

现在，我代表市人民政府，向大会报告本届政府过去五年的工作，对今后五年工作提出建议，请予审议，并请列席会议的政协委员和其他同志提出意见。

一、过去五年工作的回顾

五年来，在自治区党委、政府和市委的正确领导下，在市人大及其常委会、市政协的监督支持下，我们紧紧依靠全市人民，坚持以邓小平理论和“三个代表”重要思想为指导，认真贯彻落实科学发展观，按照“六靠”工作思路，深入开展“三百工程”项目建设大会战，解放思想，真抓实干，圆满完成了市三届人大一次会议确定的各项目标任务。与2005年相比，全市地区生产总值由222.8亿元增加到2010年的544.7亿元，年均增长14.1%；财政收入由17.05亿元增加到40.02亿元，年均增长18.6%；规模以上工业增加值由34.05亿元增加到168.7亿元，年均增长28.4%；全社会固定资产投资由129.4亿元增加到385.3亿元，年均增长24.4%；城镇居民人均可支配收入由7642元增加到15531元，年均增长15.2%；农民人均纯收入由2693元增加到5289元，年均增长14.5%；社会消费品零售总额由91.4亿元增加到209.54亿元，年均增长18%；进出口总额由7777万美元增加到1.74亿美元，年均增长17.4%。全市经济社会呈现又好又快发展的良好局面。

──过去的五年，是国民经济快速发展，产业结构不断优化的五年。五年来，我们始终坚持加快发展不动摇的理念，转变发展方式，强力推进贵港经济快速发展。三次产业比重由2005年的29.6∶32.6∶37.8调整为2010年的19.8∶45.6∶34.6，二产的比重提高了13个百分点，产业结构得到了优化。工业实力明显增强。与2005年相比，工业化率由0.91提高到2.02，工业对经济增长的贡献率达53.2%，提高了5.7个百分点，工业增加值由60.2亿元增加到218.78亿元，年均增长22.2%；规模以上工业企业达到415家，新增254家，年产值超亿元企业达到102家、新增84家，其中超10亿元企业达到9家、新增8家。5年共创中国名牌产品、中国驰名商标和广西名牌产品28个。建材、电力、冶金等支柱产业不断壮大，支撑作用进一步显现。现代农业稳步发展。全市农林牧渔业总产值由2005年的108.23亿元增加到2010年的180.91亿元，年均增长5.8%。粮食连续五年增产丰收。全市无公害农产品生产基地认定面积达233.5万亩，建成标准化畜禽养殖小区35个。市级以上产业化龙头企业发展到93家、新增84家，农民专业合作社714家、新增694家。农机化作业综合水平达到38.21%。第三产业加速发展。第三产业增加值由81.2亿元增加到188.35亿元，年均增长13.5%。苏宁电器、北京华联等一批大型商贸物流企业落户贵港，建材、汽车摩托车城等一批专业市场建成并运营。房地产、旅游、金融保险等现代服务业快速发展，城市商贸流通服务功能增强。全市接待游客总数和旅游总收入年均分别增长16.4%、32.7%。

──过去的五年，是基础设施投入大幅增加、城乡面貌焕然一新的五年。五年来，我们不断加大城乡基础设施建设力度，基础设施日趋完善。中心城区建成区面积由2005年的38.8平方公里扩大到51.7平方公里，人口由34.29万增加到50万。五年累计投入40多亿元，实施了东湖综合改造、堤路园等重点工程，建成马草江公园、迎宾大道北延段、荷城路西延段、城西污水处理厂等一批市政工程项目，完成了580多条小街小巷改造。城区污水集中处理率、生活垃圾无害化处理率由2005年的零处理分别提高到2010年的75%、100%。城区新增绿地138.9公顷，人均公共绿地面积达到9平方米。坚持城乡统筹，加强县城和中心镇、重点镇建设，大力推进新农村建设。2010年城镇化率达37.8%，比2005年末提高10.5个百分点，农村生产生活条件不断改善。综合交通网络加快完善。港口规划建设取得重大进展，《贵港港总体规划》获得了国家交通部和自治区政府批复实施。五年累计投资57.3亿元建设港口、水运等基础设施工程，新增千吨级以上泊位21个，新增港口年货物吞吐能力1800万吨；贵港至梧州航道通航等级由三级提升到二级；累计完成货物吞吐量1.51亿吨，年均增长13.7%。贵港港已跻身全国内河主要港口十强行列。南广高速公路（贵港段）建成通车，贵港至梧州高速公路、南广高速铁路（贵港段）等一批重大交通项目开工建设，实现了乡乡通油路或水泥路、村村通公路。投入33亿元用于电网建设，建成500千伏逢宜变电站等一批重点项目。投入16.4亿元用于水利设施建设，完成了53座水库除险加固任务，并兴建了一批水利基础设施工程，新增和恢复有效灌溉面积12.9万亩。投入4.2亿元实施农村饮水工程387处，解决了90.71万人安全饮水问题。投入6亿元用于水库移民基础设施和生产开发项目建设，受益群众达30万人。注重生态建设和环境保护，森林覆盖率提高到45.3%，全面完成了节能减排任务，人居环境空气质量保持国家二级标准。

──过去的五年，是改革开放稳步推进、发展活力显著增强的五年。五年来，我们坚持把科学发展的思想贯穿于全部工作的始终，用改革创新的办法解决发展中的问题。着力完善国有资产监管体系，提升国有资本运营能力。稳步推进农村综合改革，基本完成集体林权制度主体改革任务。深入推进部门综合预算、国库集中收付、政府采购和乡财县管改革，公共财政管理体系进一步完善。平稳实施公务员工资制度改革和义务教育阶段教师绩效工资改革。文化、水利、粮食流通、医药卫生、供销社等体制改革顺利推进。市县区政府机构改革顺利完成，乡镇机构改革有序开展。投融资改革不断深化，小额贷款、中小企业融资担保、政策性农业保险试点等工作扎实推进。

坚持开放合作和招商引资上项目。五年累计争取得到扩内需项目896个、资金35亿元；签约合作项目1250个，累计引进市外资金600多亿元，先后引进了华润、台泥、印尼爱凯尔、史丹利等一批知名企业和温氏集团、巨东集团、天福集团等一批农业产业化龙头企业。“三百工程”项目建设大会战成效显著。5年全市累计新开工规模以上项目1702个，台泥一、二期，华润（平南）水泥二、三期，贵港电厂一期等一批重点项目相继竣工投产，大藤峡水利枢纽工程、华电二期等重大项目前期工作取得新突破，多年为之努力的一批重大项目得到落实，增强了发展后劲。对外开放取得新成绩。五年累计实际利用外资5.7亿美元，是“十五”时期的5.6倍；2010年进出口总额达1.74亿美元，是2005年的2.23倍。

──过去的五年，是社会事业全面进步、发展势头日益向好的五年。五年来，我们始终以人的全面发展为目标，加大公共财政投入，统筹社会事业发展，公共服务能力进一步增强。深入实施科教兴市战略，全面完成了第三、第四轮创新计划目标任务，研制开发工业新产品95个，引进推广农业新品种106个，获得授权专利376件。坚持优先发展教育，五年累计投入教育经费97.84亿元，年均增长34%。义务教育经费保障机制全面建立，城乡义务教育学校教师绩效工资和学生学杂费免除等政策落实到位；投入经费7.91亿元新建、扩建或改建中小学校舍58.77万平方米，消除各类危房34.57万平方米；着力巩固“两基”攻坚成果，通过了国家级“普九”达标验收；贵港职业学院顺利搬迁，职业教育攻坚成效显著。扎实推进医改工作，加大医疗卫生基础设施投入，着力完善县级城区医院服务功能，实施了93个农村卫生机构项目，城乡公共卫生体系进一步健全，城乡医疗救助体系不断完善。文化体育事业繁荣发展。开工建设市博物馆和市图书馆，新建34个乡镇综合文化站、85个村级公共服务中心、690家农家书屋，20多个文艺作品获得自治区级以上奖项。文化遗产保护开发工作得到加强。广播电视“村村通”工程加快推进，广播电视综合覆盖率达96.5%。群众健身活动蓬勃开展，竞技体育水平得到新提高。深入推进精神文明创建活动，市民素质和城市文明程度进一步提升。人口计生工作取得新突破，人口自然增长率控制在10‰以内，计划生育率达到85%以上。国防动员、国防后备力量建设和“双拥”工作进一步加强。妇女、儿童以及残疾人合法权益得到保障。统计、民族、宗教、档案、外事侨务、对台、公积金、地方志、防震减灾等工作都取得了新的成绩。

──过去的五年，是人民生活不断改善、发展成果催人奋进的五年。五年来，我们始终以人民群众的根本利益为出发点和落脚点，着力解决人民群众最关心、最直接、最现实的利益问题，让广大人民群众共享改革发展的成果。就业和社会保障工作成效明显。五年累计新增城镇就业7.6万人、再就业2万人，转移农村劳动力23.07万人，城镇登记失业率控制在4%以内。进一步完善了社会保障体系。全市社会保障投入由2005年的1.9亿元增加到2010年的6.4亿元，年均增长27.1%。连续五年提高企业退休人员养老金标准。加强社会保险扩面征缴，社会保险覆盖总人数由2005年的51.1万人次扩大到124.5万人次。解决了政策性破产国有企业以及关闭破产集体企业退休人员、困难企业职工等群体的养老保险、医疗保险问题和村干部养老统筹问题。新型农村合作医疗参合率达92.25%，新型农村养老保险试点有序推进。进一步完善以助困、助贫、助学为重点的社会救助机制，累计筹集10.9亿元用于资助各类社会困难群体，城乡低保实现应保尽保。加大廉租住房保障力度，投入4.4亿元建设保障性住房27.9万平方米；给6748户城市低收入家庭累计发放廉租住房租赁补贴资金1020.7万元。扶贫攻坚成效明显，11.2万农民实现脱贫。建成农村沼气池3.79万个，改造农村危房4725户。认真落实优抚安置、残疾人、高龄老人优待政策和最低工资制度，把改革发展成果更多地惠及人民群众。大力实施“维稳基石工程”，建立健全了县、乡、村三级维稳工作平台;认真实施“五五”普法规划，加强和改进信访工作，着力解决人民群众反映强烈的热点难点问题；深入推进平安贵港创建活动，组织开展了“打黑除恶”等专项斗争，社会治安明显好转；高度重视应急管理、食品药品监管和安全生产工作，保持了社会大局的和谐稳定。

──过去的五年，是依法行政日益加强、发展能力明显提升的五年。在推进经济社会发展的过程中，我们高度重视政府自身建设，努力用务实的工作来落实各项政策，政府执行力和公信力不断提高。坚持依法行政，进一步规范政府工作程序，修订了《贵港市人民政府工作规则》，健全完善了管理制度和工作流程，逐步完善了重大事项集体决策、专家咨询、社会公示和市民听证等制度。自觉接受人大和政协的监督，注意听取各民主党派、工商联和社会各界的意见，5年共办理人大议案和建议347件、政协委员提案1276件。切实加强机关作风效能建设，组织开展政风行风评议和“千人评机关、百企评科室”活动。认真开展行政许可项目和规范性文件清理工作。全面实施了政府信息公开和政务公开。逐步完善了目标绩效考核机制，加强了对重要工作和重点项目的检查落实。严格落实党风廉政建设责任制，全面推进惩治和预防腐败体系建设，强化行政监察和审计监督，坚决纠正损害群众利益的突出问题，严肃查处了一批违法违纪案件，政风建设取得新成效。

五年来，我们相继荣获广西市容环境综合整治“南珠杯”竞赛B类城市特等奖、广西园林城市、自治区文明城市、自治区卫生城市、自治区人口和计划生育目标管理责任制先进单位“四连冠”、全国双拥模范城“六连冠”和自治区双拥模范城“八连冠”等荣誉称号，多项工作实现创新突破，走在了全区的前列。桂平市和覃塘区被评为“广西科学发展十佳县”，五个县市区均荣获全区科学发展进步奖。

各位代表，五年的发展令人鼓舞，五年的成就来之不易。五年来，我们克服重重困难，应对种种挑战，实现了国民经济和社会事业的跨越式发展，广大人民群众得到更多实惠，社会和谐稳定。我们所取得的每一项成绩，都离不开自治区党委、政府和市委的正确领导，离不开市人大、市政协的大力支持，更离不开全市人民的团结奋斗、顽强拼搏和辛勤劳动。在此，我代表市人民政府，向奋战在全市各条战线的广大干部群众和离退休老同志，向各民主党派、工商联和人民团体，向驻贵中区直单位和驻贵部队、武警官兵，向所有关心、支持、参与贵港改革开放和现代化建设的各界人士，致以崇高的敬意和衷心的感谢！

在充分肯定成绩的同时，我们也清醒地看到，尽管全市经济社会有了长足发展，但经济总量小、人均水平低、产业结构不优、节能减排压力大的现状还没有根本改变；产业园区没有充分发挥效应，缺乏支撑和带动力强的大产业、大企业、大项目的现状还没有根本改变；财政收支矛盾突出、城乡基础设施建设滞后、公共服务水平较低、社会管理手段落后的现状还没有根本改变。在政府自身建设方面，少数部门和一些工作人员的自身素质、办事效率还不适应加快发展的需要；政府的工作机制、行政效能有待进一步创新和提升。对这些突出矛盾和问题，我们将在今后的工作中认真研究，着力解决。

二、抢抓机遇，奋力打造西江流域核心港口城市

未来五年是贵港打造西江流域核心港口城市的关键时期、也是参与西江经济带开发建设的攻坚时期。面对复杂多变的国内外经济形势，尤其是西江流域城市之间的竞相发展，我们既有许多加快发展的有利条件和因素，但也存在着巨大的压力和困难。基础差、底子薄、经济总量小、人均水平低是贵港发展存在的最大问题。解决贵港发展中的困难和问题的根本途径是加快发展和科学发展。形势逼人，不进则退、不快也退，加快发展和科学发展是贵港当前最大的政治、最硬的道理和最紧迫的任务。因此，我们必须坚持发展第一要务不动摇，以科学发展观为统领，解放思想，实事求是，团结拼搏，励精图治，艰苦创业，多做为贵港发展打基础、谋长远的事，多做经得起历史检验的事，多做为贵港老百姓谋福祉的事。用我们扎实的行动、辛勤的汗水、富有成效的工作，努力开创贵港经济社会发展新局面。

根据中国共产党贵港市第四次代表大会精神，按照《贵港市国民经济和社会发展第十二个五年规划纲要》提出的目标任务，今后五年政府工作的总体要求是：以邓小平理论和“三个代表”重要思想为指导，深入贯彻落实科学发展观，以科学发展为主题，以转变发展方式为主线，以西江经济带建设为主战场，以改革开放为动力，以改善民生为根本，深入实施港口、园区、城市互动发展战略，统筹推进新型工业化、城镇化与农业产业化，奋力打造优势突出、辐射面广、经济发达、生态良好、文明和谐的西江流域核心港口城市，努力实现富民强市新跨越。

今后五年我市经济社会发展的主要奋斗目标是：到2015年，全市地区生产总值达到970亿元，年均增长13%，力争突破1000亿元；财政收入达到80亿元，年均增长15%，力争突破100亿元；全社会消费品零售总额达到440亿元，年均增长16%；外贸进出口总额达到3.5亿美元，年均增长15%。人口自然增长率控制在9‰以内,城镇登记失业率控制在4.5%以内。全面完成自治区下达万元生产总值能耗、二氧化碳排放量、主要污染物排放量等指标。

──综合实力上新台阶。到2015年，力争全市地区生产总值、财政收入、全社会固定资产投资、社会消费品零售收入、进出口总额、城镇居民人均可支配收入、农民人均纯收入等主要指标分别比2010年“翻一番”，进入广西城市发展先进行列。

──基础设施日臻完善。实施港口、园区、城市互动发展战略，进一步加大交通网络、产业园区和城乡基础设施建设力度，力争到2015年初步形成较为完善的港口、铁路、公路、水路等现代综合交通网络，较为完备的产业园区功能配套和城镇基础设施体系，为打造西江流域核心港口城市提供强有力的基础支撑。

──产业布局科学合理。按照“构建大港口、建设大平台、打造大产业、发展大物流”的原则，科学布局产业，认真实施项目，到2015年初步形成能源、建材、冶金、糖纸和农林产品深加工等五大主导产业与现代物流业、旅游业相结合的临江产业集群，纳税超千万元企业个数比2010年翻一番以上。

──项目建设重大突破。深入实施“三百工程”项目建设大会战，努力在重大项目建设上有新突破。力争到2015年，全社会固定资产投资突破1000亿元，年均增长20%以上。

──生活水平显著提高。到2015年，城镇居民人均可支配收入达到27300元，年均增长12%；农民人均纯收入力争突破10000元，年均增长13.6%。就业更加充分，城乡社会保障体系进一步完善，收入分配格局更加合理，人民物质文化生活更加丰富，生活质量和生活水平不断提高。

──公共服务均衡提升。力争到2015年，全市人民受教育程度稳步提高，城乡公共卫生和医疗服务水平显著改善，公共文化设施覆盖农村面进一步拓宽，公共产品和公共服务的覆盖范围进一步扩大、质量进一步提高、效能进一步提升，不断满足人民群众过上幸福美好生活的新期待。

──社会管理创新有效。力争到2015年实现社会管理能力明显增强，社会管理手段和公共安全应急处置能力不断创新和提高，社会治安防控体系不断完善，社会矛盾多元化调处机制进一步健全，社会更加和谐稳定，为广大市民创造更为优良的宜居环境。

为了实现上述目标，我们必须努力做好以下六方面工作：

（一）全力推进西江经济带建设，奋力打造经济竞争新优势

自治区党委关于打造亿吨黄金水道、建设西江经济带的决策，为贵港经济发展提供了千载难逢的机遇。我们一定要解放思想，勇于创新，开拓奋进，争当西江经济带开发排头兵。通过五年努力，发展沿江经济，做大经济总量，提高人均水平，为建设西江流域核心港口城市打牢经济基础。

加速构建现代大港口。严格实施贵港港总体规划，科学有序开发利用沿江岸线资源。重点推进苏湾作业区、郁水作业区、东山多用途泊位、桂平棉宠作业区一期、武林港二期等十大公共性港口码头建设，配合推进大藤峡水利枢纽、贵港至南宁2000吨级航道、贵港至梧州3000吨级航道、桂平至来宾2000吨级航道、贵港航运枢纽二线船闸等建设，进一步提高港口承载能力。加快完善口岸查验设施，健全口岸便捷通关模式。突出抓好港区集疏运体系建设，力争到2015年全市港口货物吞吐能力达7200万吨，集装箱吞吐能力达40万标准箱。全面推进以高速公路、铁路、内河航道为重点的综合交通运输体系建设。在加强港口基础设施建设的同时，全力配合推进梧州至贵港、桂平（石龙）至来宾、贵港至合浦、荔浦至玉林、柳州至梧州、桂平（石龙）至宾阳高速公路（贵港段）和南广高速铁路、柳州至肇庆铁路（贵港段）建设。加强国省干线公路、农村公路的建设和养护，基本实现乡乡通三级以上公路。到2015年，初步把贵港建设成为西江流域运力最大、港口优势突出、内畅外联、换乘便捷的交通枢纽中心。

加快建设产业大平台。按照集群式发展、产业链延伸、专业化分工的原则，高起点定位、强力推进贵港国家生态工业（制糖）示范园区、市江南产业园、贵港（台湾）产业园、桂平长安产业园、平南产业园等五个重点产业园区的规划、建设和管理。着力创新完善园区管理体制、运行机制、监督机制、考核机制和激励机制，扩大园区自主权，强化服务功能，提高办事效率，使园区成为高效运转、规范有序、自我积累、自主发展的实体。从合理布局、加速发展和做大做强的目标出发，把五大园区科学规划为15个功能分区，每个功能分区培育2-3个主导产业，每个主导产业培植1-2个龙头企业。围绕功能分区定位，统筹配置资源要素、统筹建设基础设施、统筹布局产业项目、统筹落实扶持政策，努力实现园区内企业的有效配合和协作配套。要制定比周边地区更加优惠的政策，营造更加优良的环境，重点吸引世界500强、全国500强、区内100强企业落户园区。争取国家和自治区支持设立以承接粤、港、澳和珠三角产业转移为重点的贵港保税港区以及建设西江经济带的优惠政策。力争5年内园区工业产值占全市工业的60%以上，把产业园区建设成为贵港加快发展的重要载体。

着力打造临江大产业。按照科学谋划、适度超前的要求，进一步调整优化全市产业布局，以做强企业为手段，以做大产业为目标，推进临江产业集聚。贵港国家生态工业（制糖）示范园重点发展糖纸循环、热电联产、电子信息、节能环保新材料等产业；市江南产业园重点发展羽绒、轻纺、皮革、物流等产业；贵港（台湾）产业园重点发展建材、现代物流、船舶修造、农林产品加工等产业；桂平长安产业园重点发展机械制造、生物化工、陶瓷制品、服装加工等产业；平南产业园重点发展建材、机械、电子、生物科技等产业。充分发挥区位优势，以非常办法、非常措施、非常力度、非常政策谋划和推动工作，加快形成以能源、建材、冶金、糖纸和农林产品深加工五大主导产业与现代物流相结合的临江产业集群，促进沿江经济的快速发展。

积极培育区域大物流。加强物流基地建设，推动现代生产性和生活性服务业发展，把贵港建设成为区域性商贸物流中心。重点规划建设罗泊湾现代物流园、东津现代物流园、桂平长安物流园等十二个物流园和钢材、水泥、煤炭、林产品、禽畜、粮糖、陶瓷等十大专业市场或交易中心，建设一批集仓储、配送、电子商务、商品加工为一体的现代物流基地，努力实现贵港港从单一的中转仓储基地向商贸型、综合型现代化物流基地转型。优先引进物流大企业，吸引国内外强优物流企业入驻贵港，带动中小物流企业发展。积极拓展物流新业态，鼓励和支持工贸企业分离物流服务业务，发展第三方物流。鼓励发展现代金融、研发设计等生产性服务业，实现先进制造业与现代服务业融合发展，延伸物流产业链。到2015年，力争培育一批经营服务先进、竞争力较强的现代物流龙头企业，初步建成西江流域商贸物流中心。

（二）完善城市功能，打造宜居生态园林城市

统筹港口、园区、城市互动发展，坚持城乡并重、建管并举的理念，按照高起点规划、高标准建设、高水平管理的原则，加快城镇开发建设和管理步伐，叫响“南国荷城、岭南古郡、千年良港”三大品牌，着力打造现代化港口城市。力争到2015年，全市城镇化率达45%以上。

推进中心城区建设。按照新一轮城市总体规划定位，高起点完善城西片区、港口新区、城北新区二期、郁江北堤片区、新火车站片区、江南片区等六大组团控制性详规编制和城市设计方案。城西新区定位为集商业、商务、居住和旅游四位一体的城市滨水生态综合新区，着重规划建设沿迎宾大道两侧的大型商务高楼群，打造商业商务新区。港口新区定位为港口物流、加工贸易等临港产业承载地，设施配套完善的活力临港新区。城北新区二期要突出高标准打造金融、商务、休闲娱乐、文化体育等标志性空间和载体，增强对各类要素的吸引力和集聚力。郁江北堤片区定位为商贸、居住、休闲区，重点加大旧城区商业中心改造力度，力争完成东湖和堤路园综合改造工程，重塑旧城核心商圈功能和形象，将其打造成为郁江十里黄金水岸和贵港市的标志性工程。新火车站片区着重规划建设商住、物流项目，打造城市窗口新形象。江南片区要紧密结合产业园区和港口规划建设，加快完善服务配套功能，推动园城一体化发展，打造物流加工新城和统筹城乡发展的示范区。高标准启动实施市区路网建设大会战，规划建设西南大道、仙衣路南延段、城南大道、城东大道、滨江大道等主干路，完善新老城区组团的主干路网络。开工建设同济大道、友谊大道、江南大道和同济大桥、西南大桥，完成东环郁江特大桥工程，缓解过江交通压力。完成解放路北延段、建设路西延段、中山路中段等道路改造，改善市区交通拥堵状况。建成市博物馆、档案馆、体育中心和文化艺术中心，新建民族文化公园、南湖公园等休闲景区。继续实施以城市绿化、亮化、美化、净化为内容的城市主次街道街景改造工程。凡新建或改建道路，都综合配套交通标识、绿化亮化和地下管网；凡新建大型住宅小区，都必须综合配套教育、医疗、商场、休闲健身、社区管理中心等公共设施，让市民生活更安全、更便捷、更舒适。

提高城市管理水平。按照“重心下移、条块结合、以块为主”的要求，创新和完善与现代化城市相适应的管理体制，形成合理分工、相互协调、高效管理的工作格局。进一步创新城市管理机制，整合管理队伍、落实责任、依法管理，逐步建立和完善城市规划建设、公共服务、市容市貌、城市交通、园林绿化、环境卫生等长效管理机制，提高管理效能。继续深入实施“城乡清洁工程”，重点治理交通混乱、占道经营、市容卫生、环境污染等突出问题，坚决打击违规违法用地建设行为，努力营造优美、亮丽、有序的城市环境。

加强县城和城镇建设。高标准规划和建设城镇，完善市政基础设施和公共服务功能，有效增强县（市、区）城区承载力。围绕推进扩权强县、扩权强镇和打造主城区卫星城镇，完成所有村镇的规划编制，实现城乡规划全覆盖。突出抓好西江沿岸和贵梧高速公路沿线的12个人口规模较大的重点镇建设，着力拓展框架，完善功能，提高水平，形成特色。积极推进户籍制度管理改革，有序引导人口、产业向县城和中心镇集中，促进城镇化与新农村建设的良性互动。

提升城市商贸服务水平。适应区域性中心城市发展的需要，合理规划布局中心城区商务区、特色商贸步行街、星级酒店，扩大城市经济容量。大力发展金融、保险、证券、信托投资、中介、信息等现代服务业。培育发展总部经济，支持有实力企业在贵港成立结算和研发中心。高度重视城市居民生活消费需求，积极发展生活服务业，加快形成与城市功能相适应的服务业体系。加强农村流通体系和服务网络建设，因地制宜建设综合性或专业性的农副产品和其他产品批发市场，继续推进“万村千乡市场工程”和“新农村现代流通网络工程”，有效拉动城乡消费。依托区位和资源优势，加大旅游开发、建设和宣传推介的力度，带动服务业繁荣发展，加快把贵港建成广西乃至全国知名的宗教文化生态旅游城市。

（三）强化工业主导核心战略，着力提升产业竞争力

坚持工业立市战略不动摇，以产业园区为载体，加速产业聚集，做大工业总量，全面提高工业经济的整体水平。到2015年，力争规模工业增加值突破500亿元，年均增长20%以上。

突出抓好五大产业。按照“发展优势产业，打造核心企业，延伸产业链条，培育产业集群”的思路，完善产业发展规划，大力调整优化产业结构。重点抓好能源、建材、冶金、糖纸和农林产品深加工五大产业的培育、巩固和壮大，使之成为贵港财政的重要来源。力争到2015年能源产业集群产值达150亿元，建材产业集群产值达200亿元，冶金产业集群产值达150亿元，糖纸产业集群产值达100亿元，农林产品深加工产业集群产值达150亿元。同时，还要紧紧抓住国家“十二五”支持战略性新兴产业发展的重大机遇，加大对新兴产业的招商力度和财政扶持力度，大力发展生物化工、机械制造、电子信息、船舶修造等加工制造产业以及新能源、新材料等战略性新兴产业，推动结构调整、产业升级，加快形成具有较强竞争优势的贵港特色工业体系。

扶优扶强龙头企业。坚持以做强存量、做大增量为重点，鼓励骨干企业通过技术创新、开拓市场、靠大联强、低成本兼并扩张等措施，培植一批主业优势明显、核心竞争力强的龙头企业。力争到2015年，年产值超亿元企业达250家以上，其中超10亿元企业30家以上。大力扶持中小企业和微型企业提档升级，促进中小企业与大型企业协作配套、集群发展。力争5年内新增规模企业300家以上，努力培育一批纳税支柱企业。

大力发展循环经济和节能经济。鼓励发展绿色经济和循环经济，积极开发和使用绿色清洁能源，提高资源循环利用和废弃物综合利用水平。积极推动现有企业实行循环经济改造，扩展电力、建材、糖纸、农产品加工等循环产业链。重点加快推进广西（桂东南地区）循环经济环保产业示范基地建设，实现以垃圾焚烧发电为核心的多种废弃物综合处理和循环利用，争取建设成为全国中等规模城市资源循环利用的示范基地。严格执行环评审批和固定资产投资项目节能评估和审查制度，提高项目环境准入条件，积极引进科技含量高、附加值高、能耗低、污染少的企业。组织实施重大节能技术专项行动，大力推广应用高效节能新技术、新工艺、新设备和新产品。

（四）突出富民增收，推进城乡一体发展

以农民增收为核心，加快推动先进生产要素向农村流动、基础设施向农村延伸、公共服务向农村覆盖、现代文明向农村传播，构建城乡一体发展格局。到2015年，粮食总产稳定在145万吨以上，农业增加值达到130亿元，年均增长6%以上。

加快发展现代农业。积极发展农业产业化，促进农业发展方式转变。稳定发展粮食生产，确保粮食安全。实施浔郁平原农机化推进行动，加快农业机械化进程。抓好农户“万元增收工程”和“菜篮子”工程，大力发展城郊型农业，提高城市副食品供应保障能力。加强农业产业结构调整，加大扶持发展特色农业力度，加快形成一批规模相对集中的现代、高效、生态农业生产基地。力争5年内新增标准化畜禽小区100个、水产养殖小区20个、特色种植业基地25万亩。积极推进农业产业化经营和规模化生产，引导农民专业合作社健康发展，培育一批产业规模大、带动辐射能力强的种养型、加工型、流通型龙头企业。力争到2015年，市级以上农业产业化龙头企业达120家以上，年销售收入超亿元龙头企业70家以上，其中超10亿元企业6家以上；培育发展1000家以上农业产业化经营组织，农户专业化生产率达60%以上，形成 50 个以上特色效益突出的专业村（屯），农林产品深加工产业集群产值达150亿元以上，逐步把贵港建设成为广西重要的农产品生产加工基地和高效现代农业的示范基地。

加强农村基础设施建设。加大政府投入，五年内完成所有病险水库和15座重点大中型病险水闸的除险加固，基本完成10个大中型灌区节水续建配套工程；加强防洪薄弱环节建设，基本完成重点中小河流重要河段治理；强化农村饮水安全工程建设，基本解决农村人口饮水不安全和困难的问题。加强农村公路网络建设，五年内基本实现建制村全部通水泥路（或柏油路）的目标。实施新一轮农村电网升级工程，提高农村供电能力。健全完善农技推广体系，加强农产品质量安全监管、气象水文预报、山洪地质灾害预警、森林防火、动物防疫、农林有害生物防控等体系建设，提高综合防控能力。

推进城乡统筹发展。强化乡村规划，加强农村文化、教育、卫生等基础设施建设和管理，逐步实现城乡基本公共服务均等化。深入实施“城乡风貌改造”工程，着力改善村容村貌。大力发展“一村一品”、“一乡（镇）一业”特色种养和“农家乐”休闲产业，强化农村产业支撑。切实抓好新一轮扶贫开发和整村推进工作，继续加强库区移民基础设施建设。认真落实粮食、良种、农机、家电下乡等各项强农惠农政策，完善财政支农资金稳定增长机制，增加农民政策性收入。加强对农民的职业技能培训，提高农民职业技能和创收能力，促进农民转移就业，增加农民务工收入。

（五）进一步深化改革扩大开放，不断激发经济社会发展活力

积极推进观念创新、体制机制创新和管理创新，为经济社会发展提供强大动力。实施更加积极主动的开放战略，聚合发展要素，推动全市经济总量加速扩张、质量加快提升。

深入推进各项改革。健全国有资产监督管理体制，提升国有资本运营水平。继续深化财政管理体制改革，健全以部门预算、国库集中收付、非税收入收支两条线、政府采购、政府投资评审、支出绩效评价为重点的管理制度，提高财政科学化精细化管理水平。加大政府购买社会服务力度，推行政府投资项目代建制。加强政府债务管理，切实防范财政风险。依法加强税收征管，加大社会综合治税力度。积极稳妥地推进资源性产品价格改革，加强资源要素市场化配置。继续深化行政管理体制改革，进一步理顺部门职责权限。积极稳妥推进事业单位分类改革，逐步实现政事分开、事企分开和管办分离。加快推进扩权强县、扩权强镇，强化政策支持，增强县域经济自主发展活力。深化农村综合配套改革，积极促进土地承包经营权合理有序流转。全面推进集体林权制度配套改革，进一步激发林业发展活力。完善农村金融服务，扩大农业保险范围。鼓励发展农村商业银行、村镇银行、小额贷款公司、融资性担保公司，大力引进市外金融机构、担保机构和股权投资机构。加强企业上市培育，扩大企业直接融资渠道和规模。加强社会信用体系建设，营造优良的信用环境。完善和落实各项优惠政策，支持民间资本进入基础设施、公用事业、科教文体卫、环境保护等领域，不断拓宽非公经济的发展空间。

扩大对外开放合作。深入实施互利共赢的开放战略，主动承接发达地区产业转移，建设国家级承接产业转移示范区。围绕产业结构升级和新兴产业培育，整合招商资源，创新招商方式，积极推进产业集群式招商。重点加强对世界500强、中国500强和广西100强企业的招商力度，着力引进关联度高、带动力强的龙头型、基地型项目。通过多种渠道和形式，开展各种丰富多彩的活动，进一步扩大对外交流与合作。强化出口产业集群和出口基地建设，切实增强出口产品市场竞争力，努力提高对外开放合作水平。

（六）致力民生改善，推动基本公共服务均等化

经济发展的根本目的在于保障和改善民生，增进人民的福祉。为顺应人民群众过上更加美好生活的新期待，我们必须加快建立健全基本公共服务体系，实现基本公共服务均等化，不断提高城乡群众生活水平。

大力发展社会事业。坚持教育优先战略，认真落实中长期教育改革和发展规划纲要，努力促进各类教育优质、均衡、特色化发展。实施新一轮中小学校布局调整，加快推进城区学校新建和改扩建工程，加大对农村和薄弱学校的建设力度，改善城乡办学条件。重视发展学前教育，提升义务教育水平，逐步普及高中阶段教育，建立特色职业教育，加快贵港高等院校建设。鼓励和支持民间资本以独资、股份、合作等多种形式兴办教育。加快深化医药卫生体制改革，加强公共卫生服务体系建设，建立覆盖城乡居民的基本医疗卫生服务网络。5年内中心城区新建一所综合医院和精神卫生医院，改造或新建一批乡镇、社区卫生服务设施，不断满足群众对医疗服务的需求。支持中医药事业发展，抓好重大传染病、职业病防治。启动实施第五轮创新计划，加快建设以企业为主体、市场为导向、产学研相结合的技术创新体系，增强企业技术创新能力。深化文化体制改革，积极培育文化产业。立足贵港人文特色，大力挖掘和传承历史文化，打造优秀文化精品。加强农村广播影视公共服务体系建设，扩大广播电视“村村通”的覆盖面。加大文化基础设施投入，推进文化惠民工程。5年内新建一批村级公共服务中心和农家书屋，推动公共文化设施向乡村覆盖。大力开展全民健身运动，增强全民体质。建立健全计划生育利益导向机制，大力推进诚信计生，统筹解决人口问题。抓好市职工之家、社会福利中心和残疾人康复设施建设。进一步加强国防动员、国防后备力量和人民防空工作。认真实施妇女儿童发展规划，支持妇女、共青团、工会等人民团体开展工作。统筹推进统计、民族、宗教、外事侨务、对台、公积金、地方志、防震减灾等各项事业发展。

完善就业和社会保障体系。坚持把就业作为民生之本，多渠道开发就业岗位，力争5年内新增城镇就业6.5万人，新增农村富余劳动力转移21.5万人。完善以基本养老、基本医疗为重点的社会保险体系，全面落实失地农民保障制度，“十二五”期末实现城乡居民社会养老保险全覆盖。加大扶贫济困力度，提高城乡低保、农村五保等保障水平。建立社会救济和社会保障与物价上涨挂钩联动机制。大力推进养老服务体系建设。加大以公共租赁房为主的保障性住房供给力度，稳步推进廉租住房实物配租。五年新建10000套以上保障性住房，完成农村危房改造3万户以上，努力为城乡中低收入家庭提供住房保障。

加强生态环境建设。把生态建设列入政府的重点工作抓紧抓好，严格环保准入，决不能以牺牲生态环境来换取一时的发展。切实做好重要水系的保护和治理，强化水源地、城市取水口等重点地区的环境保护。规划建设中心城区第二饮用水源工程，提高农村自来水普及率。加强城镇污水、生活垃圾、固体废弃物处理设施的建设和运行管理，全面实施“村收集、乡转运、县处理”的垃圾处理模式，改善城乡环境。加强农村户用沼气建设，治理农村面源污染，新建沼气池2万个。大力开展植树造林，积极实施“山上调结构提质量、山下扩绿化增总量”为重点的“绿满八桂”造林绿化工程。实施最严格的耕地保护制度，严格依法依规管地用地，集约节约使用土地。继续开展矿产资源开采秩序整治，切实做好关闭矿山生态恢复工作。

加强和创新社会管理。把创新社会管理体制机制、完善社会管理格局、加强社会管理能力建设作为一项紧迫任务，推进由管理型政府向服务型政府转变。加快建立健全维护群众权益机制、行政决策风险评估和纠错机制。加强信访、人民调解、行政调解、司法和仲裁调解工作，拓宽社情民意表达渠道，主动化解社会矛盾。创新城乡新型社区管理体系和服务体系，进一步加强安置帮扶、社区矫正和青少年事务工作，加强特殊人群和流动人口服务管理。充分发挥基层组织、政法综治组织以及工青妇等群团组织、行业协会和商会等社团组织在社会管理中的重要作用。全面推进企业工资集体协商等制度，积极构建和谐劳动关系。加强食品药品监管，保障人民群众饮食和用药安全。大力实施普法教育和“维稳基石工程”，提升依法管理社会和治安综合治理水平。健全公共突发事件应急管理机制，强化安全生产责任制和责任追究制，努力营造社会安定、百姓安宁的良好氛围。

三、全面提升政府执行力，确保实现五年奋斗目标

要实现今后五年的宏伟目标，既需要全市上下解放思想、求实创新、真抓实干，更需要各级政府机关和全体公务人员执行力的提升。我们一定要努力践行党的宗旨，牢固树立为人民服务的思想，努力学习为人民服务的本领，要有扎实为人民服务的行动，力求良好为人民服务的效果。对于贵港的加快发展和科学发展，我们一定要有一种等不起的紧迫感、慢不得的危机感、坐不住的责任感，不被困难所吓倒，不为非议所动摇，聚精会神搞建设，一心一意谋发展，努力把贵港打造成为西江流域核心港口城市。

解放思想，开拓创新。坚持与时俱进、更新观念，着力转变不适应科学发展的思想观念，切实摒弃满足现状、不思进取的思想，大力破除因循守旧、固步自封的思维，坚决克服惧怕困难、畏首畏尾的行为，在解放思想中真抓实干，在转变观念中破解难题。强力推进工作体制机制创新，大胆革除不利于贵港科学发展的思维定势和行为习惯，大力突破不利于贵港加快发展的条条框框和陈规陋习，按照简单、明了、易操作的要求，进一步健全完善各项工作制度和工作流程，建立健全目标、责任、考核体系，提高政府工作效率和管理水平。积极创新工作方法，注重用民主的方法集中民智，用市场的办法配置资源，用综合的手段解决矛盾。旗帜鲜明地鼓励创新、支持改革，只要有利于加快发展和科学发展、有利于改善民生、有利于社会和谐稳定、不违反政策法规，都应该大胆地闯、大胆地干，以奋发有为的精神状态，不断开创各项工作新局面。

注重学习，提升本领。要实现今后五年宏伟目标，既要有想干事的敬业精神和愿望，更要有干成事的能力和本领。要牢固树立重视学习、终身学习的理念，更加主动地学习各种新知识、增长新本领，学习和掌握国家政策法规和现代经济、科技、管理等方面的知识。提高战略思维能力，善于研判形势，把握大局，增强决策的科学性和工作的主动性、预见性。坚持问政于民、问需于民、问计于民，促进政府决策民主化、科学化，使政府各项工作体现群众意愿、符合群众利益、满足群众需求。善于学习借鉴外地先进经验和做法，善于把上级部署与贵港实际相结合，把满腔热情与务实态度相结合，把各项决策与群众利益相结合，不为过去的成绩而自满，不为既有的经验所束缚，以更宽广的视野、更有效的举措推进工作，争创一流业绩。

真抓实干，勇于担当。为民要真心、工作要实干。要坚持察实情，说实话，办实事，求实效，力戒官僚主义、形式主义。深入开展政府效能提升活动，加强机关作风建设，大力整治机关中存在的懒、散、慢、庸、拖等不良风气和不作为、乱作为、慢作为现象，努力提高政府机关和干部的执行力。切实精简会议、压缩文件、减少事务性活动，使各级领导干部把更多的时间和精力放在抓大事、解难事、办实事上。积极倡导勇于担当、敢于创造、善于作为的作风。只要是对贵港发展有利的事情，就要积极主动、敢作敢为、雷厉风行，竭尽全力把事情办好办实。凡是市委、市政府决定的事项，各级政府和部门都要坚定不移、想方设法抓落实，确保目标实现。要完善目标绩效考评体系，严格行政问责制，努力形成全体公务人员人人有激情、个个争先进的氛围。

勤政廉政，清正廉洁。严格按照法定权限和程序行使权力、履行职责，自觉接受市人大及其常委会的法律监督和工作监督，主动接受政协和各民主党派的民主监督以及人民群众和社会舆论的监督，切实做好人大代表建议、政协委员提案办理工作。健全重大问题调查研究、专家咨询和社会公示与听证及决策责任制度，保证决策科学民主、依法合规、切实可行。廉洁是一个地方发展和干部成长之福，腐败是一个地方发展和干部成长之祸。各级政府要严格落实党风廉政建设责任制，健全完善惩治和预防腐败体系，规范行政权力的运行，提高行政决策的透明度，加强行政监督和审计监督，从源头上防治腐败，以实际行动树立政府勤政廉洁的良好形象。

各位代表！时代赋予重任，人民寄予重托。今天的贵港，站在新起点，树立新目标，充满新希望。让我们倍加珍惜团结进取的好局面，倍加珍惜和谐稳定的好形势，倍加珍惜人民群众的信任和支持，在市委的坚强领导下，紧密团结和依靠全市人民，以更加坚定的信心、更加务实的作风、更加有为的举措，攻坚克难，锐意进取，为实现富民强市新跨越而努力奋斗！
